# Prevalence of Medical Cannabis Use and Associated Health Conditions Documented in Electronic Health Records Among Primary Care Patients in Washington State

**DOI:** 10.1001/jamanetworkopen.2021.9375

**Published:** 2021-05-06

**Authors:** Theresa E. Matson, David S. Carrell, Jennifer F. Bobb, David J. Cronkite, Malia M. Oliver, Casey Luce, Udi E. Ghitza, Clarissa W. Hsu, Cynthia I. Campbell, Kendall C. Browne, Ingrid A. Binswanger, Andrew J. Saxon, Katharine A. Bradley, Gwen T. Lapham

**Affiliations:** 1 Kaiser Permanente Washington Health Research Institute, Seattle; 2 Department of Health Services, University of Washington, Seattle; 3 Center for the Clinical Trials Network, National Institute on Drug Abuse, National Institutes of Health, Bethesda, Maryland; 4 Division of Research, Kaiser Permanente Northern California, Oakland, California; 5 Center of Excellence in Substance Addiction Treatment and Education, Veteran Affairs Puget Sound Health Care System, Seattle, Washington; 6 Kaiser Permanente Colorado Institute for Health Research and Colorado Permanente Medical Group, Denver

## Abstract

**Importance:**

Many people use cannabis for medical reasons despite limited evidence of therapeutic benefit and potential risks. Little is known about medical practitioners’ documentation of medical cannabis use or clinical characteristics of patients with documented medical cannabis use.

**Objectives:**

To estimate the prevalence of past-year medical cannabis use documented in electronic health records (EHRs) and to describe patients with EHR-documented medical cannabis use, EHR-documented cannabis use without evidence of medical use (other cannabis use), and no EHR-documented cannabis use.

**Design, Setting, and Participants:**

This cross-sectional study assessed adult primary care patients who completed a cannabis screen during a visit between November 1, 2017, and October 31, 2018, at a large health system that conducts routine cannabis screening in a US state with legal medical and recreational cannabis use.

**Exposures:**

Three mutually exclusive categories of EHR-documented cannabis use (medical, other, and no use) based on practitioner documentation of medical cannabis use in the EHR and patient report of past-year cannabis use at screening.

**Main Outcomes and Measures:**

Health conditions for which cannabis use has potential benefits or risks were defined based on National Academies of Sciences, Engineering, and Medicine’s review. The adjusted prevalence of conditions diagnosed in the prior year were estimated across 3 categories of EHR-documented cannabis use with logistic regression.

**Results:**

A total of 185 565 patients (mean [SD] age, 52.0 [18.1] years; 59% female, 73% White, 94% non-Hispanic, and 61% commercially insured) were screened for cannabis use in a primary care visit during the study period. Among these patients, 3551 (2%) had EHR-documented medical cannabis use, 36 599 (20%) had EHR-documented other cannabis use, and 145 415 (78%) had no documented cannabis use. Patients with medical cannabis use had a higher prevalence of health conditions for which cannabis has potential benefits (49.8%; 95% CI, 48.3%-51.3%) compared with patients with other cannabis use (39.9%; 95% CI, 39.4%-40.3%) or no cannabis use (40.0%; 95% CI, 39.8%-40.2%). In addition, patients with medical cannabis use had a higher prevalence of health conditions for which cannabis has potential risks (60.7%; 95% CI, 59.0%-62.3%) compared with patients with other cannabis use (50.5%; 95% CI, 50.0%-51.0%) or no cannabis use (42.7%; 95% CI, 42.4%-42.9%).

**Conclusions and Relevance:**

In this cross-sectional study, primary care patients with documented medical cannabis use had a high prevalence of health conditions for which cannabis use has potential benefits, yet a higher prevalence of conditions with potential risks from cannabis use. These findings suggest that practitioners should be prepared to discuss potential risks and benefits of cannabis use with patients.

## Key Points

**Question:**

Among primary care patients, what is the prevalence of electronic health record documentation of medical cannabis use and health conditions for which cannabis use might have benefits and risks?

**Findings:**

In this cross-sectional study of 185 565 patients, 2% had past-year medical cannabis use documented in their electronic health records. Among patients with documented medical cannabis use, 44.5% had documented health conditions for which cannabis use might confer benefits, and 54.5% had conditions for which it might confer risks.

**Meaning:**

These findings suggest that primary care practitioners should be prepared to discuss potential risks as well as potential benefits of cannabis use with patients.

## Teaser

This cross-sectional study describes the prevalence and clinical characteristics of primary care patients with cannabis use documented in their electronic health records.

## Introduction

Medical and recreational cannabis use is increasing in the US.^1,2^ Most US states have legal access to cannabis,^3,4^ and the perceived risk of cannabis use is decreasing.^1,5^ Many people may use cannabis to manage medical and psychiatric symptoms and sometimes to replace prescribed medications despite little understanding of the potential risks and benefits of cannabis.^6^ Cannabis use is thus highly relevant to a patient’s medical care, but how often it is addressed in health care settings with medical practitioners is unknown. Electronic health record (EHR) data can provide novel information on how often medical cannabis use is recognized and documented during outpatient encounters.

The purpose of this study was to describe the prevalence and clinical characteristics of primary care patients with medical cannabis use documented in the EHR. The study, conducted in a large health care system where primary care patients are routinely screened for cannabis use in a state where medical and recreational cannabis use is legal, used EHR data to identify patients using cannabis to manage symptoms (hereafter referred to as medical cannabis use). Specifically, the study describes the prevalence of medical cannabis use and health conditions for which cannabis use has potential benefits and risks across 3 mutually exclusive groups of patients: those with documented medical cannabis use, those with documented use of cannabis without evidence of medical use (other cannabis use), and those with no cannabis use documented in their EHR.

## Methods

### Design and Setting

This descriptive, cross-sectional study was conducted in Washington State, where medical cannabis use has been legal since 1998 and adult recreational use has been legal since 2012. The study was conducted at Kaiser Permanente Washington (KPWA), a large integrated health care system with 25 primary care clinics at the time of this study (November 1, 2017, to October 31, 2018). Kaiser Permanente Washington screens primary care patients annually for cannabis use as part of integrated mental health care.^7,8^ Data (obtained exclusively from KPWA’s Epic EHR and insurance claims) included patient demographic characteristics, diagnoses, cannabis screen results, medication fills, and EHR free-text documentation (eg, encounter notes). This study received approval and waivers of consent and Health Insurance Portability and Accountability Act authorization from the KPWA Health Research Institute Institutional Review Board. All data were deidentified. This study followed the Strengthening the Reporting of Observational Studies in Epidemiology (STROBE) reporting guideline.^9,10^

### Sample

Eligible KPWA patients were 18 years or older and completed cannabis screening at a primary care visit during the 1-year study period before data extraction (N = 193 472). We excluded patients who were also KPWA employees (n = 7900) or had opted out of having their EHR used for research (n = 7). For patients with more than 1 cannabis screen during the study (n = 14 663), we randomly selected a single screen for person-level inference.

### Measures

#### EHR-Documented Cannabis Use

##### EHR-Documented Past-Year Cannabis Use: Medical, Other, and No Use

**Figure 1.**
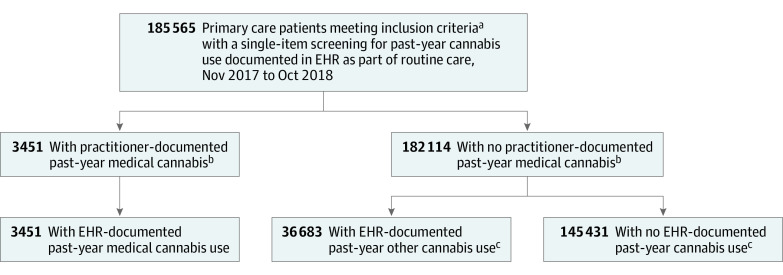
Process for Creating the Composite Measure of Electronic Health Record (EHR)–Documented Cannabis Use ^a^Excludes Kaiser Permanente Washington employees and patients opting out of inclusion of their EHR in research. ^b^As determined by natural language processing–assisted EHR review and *International Statistical Classification of Diseases and Related Health Problems*, *Tenth Revision* diagnoses. ^c^As determined by patient report of cannabis use on an annual cannabis screen.

The primary measure of past-year EHR-documented cannabis use categorized patients into 3 mutually exclusive groups: (1) medical use, (2) other use, and (3) no use (Figure 1). This composite was based on 2 binary measures: any practitioner-documented medical cannabis use based on natural language processing (NLP)^11,12^ or diagnostic code(s) for medical cannabis use (described below) and any patient-reported cannabis use from annual cannabis screening (described below). All patients with any practitioner-documented medical cannabis use were categorized as having EHR-documented medical cannabis use; other patients with any patient-reported cannabis use were categorized as having EHR-documented other cannabis use. Patients with no practitioner-documented medical cannabis use and no patient-reported cannabis use were categorized as having no EHR-documented cannabis use.

##### Patient-Reported Cannabis Use Based on Annual Screening

Primary care patients at KPWA routinely complete a 7-item behavioral health screen before meeting with their practitioner, with results documented in the EHR. The screen includes a question about frequency of past-year cannabis use adapted from a validated alcohol screen^13^: “How often in the past year have you used marijuana?” (never, less than monthly, monthly, weekly, or daily/almost daily).^14^ This measure was dichotomized to define patient-reported cannabis use (never vs responses other than never) for creation of the 3-category measure of medical cannabis use above. The cannabis screen does not ask patients to differentiate between medical and recreational cannabis use.

##### Practitioner-Documented Medical Cannabis Use From NLP or Diagnosis

We defined cannabis use documented in the EHR as medical cannabis use if the documentation indicated it was used for a health condition or symptom. To identify EHR documentation of practitioner-documented medical cannabis use, all EHR notes were evaluated for mentions of medical use in the past year or within 7 days after a patient’s cannabis screen date (ie, consistent with the past-year screen timeframe and to account for delays in documentation). To evaluate EHR notes, we had previously trained an automated machine-learned NLP algorithm to identify cannabis and cannabinoid terms (eg, *marijuana*, *cannabis*, *THC*, *CBD*, and *pot*) and to flag as nonrelevant negated (“denies cannabis use”), historical (“smoked marijuana as a teenager”), and hypothetical (“considering CBD for joint pain”) mentions. The algorithm, developed before this study,^15^ was written in Python^16^ and trained on an independent gold standard derivation sample of 1093 notes broadly representative of KPWA practitioners in which all relevant cannabis mentions had been manually identified with discrepancies resolved through consensus. The algorithm was then evaluated in an independent validation sample and achieved high specificity (94%) but limited sensitivity (67%).^15^ We therefore supplemented the automated NLP algorithm with NLP-assisted manual review to identify medical cannabis use mentions not captured by the NLP algorithm. Patients were categorized as having medical cannabis use if they had 1 or more EHR note and/or an *International Statistical Classification of Diseases and Related Health Problems, Tenth Revision (ICD-10)* diagnosis that indicated past-year medical cannabis use. Some patients (n = 964) were classified as having medical use despite reporting no past-year use on the cannabis screen, potentially reflecting practitioner documentation of CBD and other cannabinoid use not captured by the routine screen, which asks about marijuana use.

#### Other Measures

##### Health Conditions for Which Cannabis Use Has Potential Benefits or Risks

Health conditions documented in patients’ EHRs were categorized into 3 groups based on the National Academies of Science, Engineering, and Medicine comprehensive review on the health effects of cannabis and cannabinoids.^6^ Health conditions were categorized based on largely observational research as having moderate to conclusive evidence of therapeutic benefit from cannabis use (potential benefits) or having moderate to conclusive evidence of harm or risk from cannabis use (potential risks), at least for subgroups of patients.^6^ The remainder of conditions were categorized as having limited, inconclusive, mixed, or unavailable evidence (inconclusive evidence of benefit or risk). Binary indicators of health conditions were based on the presence of 1 or more *ICD-10* codes within 1 year before and/or on the cannabis screen date (codes available on request). Chronic noncancer pain was defined as documentation of 2 or more *ICD-10* codes for similar pain types at least 30 days apart or an *ICD-10* diagnosis for general chronic noncancer pain.^17^ Conditions that qualified for Washington State physician–authorized medical use at study start were also identified; although authorization was no longer required to purchase cannabis, it offered benefits (eg, larger per-visit purchases and home-based plant growth).^18^

##### Prescription Medications of Interest in Patients Using Cannabis

Practitioners may be concerned about patients’ cannabis use complicating medical treatment or in place of prescribed medications with known efficacy.^19^ Three classes of prescription medications are therefore described: (1) those that treat conditions for which cannabis may have benefit,^6,20,21^ (2) those that treat mental health or substance use disorders,^20,22,23^ and (3) those that are potentially addictive.^22,24,25^ Binary indicators of prescription medication were based on the EHR or insurance claims indicating that the medication was dispensed within 1 year before and/or on the cannabis screen date (codes available on request).

##### Covariates

Sociodemographic variables, collected from patients by KPWA and documented in the EHR before and/or at the time of cannabis screening, included age (18-29, 30-44, 45-64, or ≥65 years of age), sex (binary female or male), race (American Indian or Alaska Native, Asian, Black, Native Hawaiian or Pacific Islander, White, multiracial, or other or unknown race), Hispanic ethnicity (binary Hispanic or non-Hispanic), and insurance type (commercial, Medicare, Medicaid and subsidized, or unknown). These characteristics represent lived experiences that may influence documentation of medical cannabis use and development of health conditions. Because patients with more health care visits have increased opportunity for EHR documentation of medical cannabis use and health conditions, we created a patient-level measure for the number of days with clinical encounter notes (ie, note-days) in the past year or within 7 days after a patient's cannabis screen date. The clinic where cannabis screening occurred was also identified.

### Statistical Analysis

We estimated the unadjusted prevalence of EHR-documented past-year medical cannabis use, other cannabis use, and no cannabis use. We described unadjusted demographic and clinical characteristics across categories of EHR-documented past-year cannabis use. Using EHR-documented past-year cannabis use as the exposure of interest, we estimated the adjusted prevalence of each health condition and prescribed medication, separately, using logistic regression models.^26,27^ We adjusted for age, sex, race, ethnicity, insurance type, and the natural log of note-days to account for potential differences between patients with medical, other, and no cannabis use. In sensitivity analyses, we further adjusted for clinic to account for nonmeasured differences between clinic patients. We did not test for statistically significant differences because this was a descriptive study.^28^ We used 95% CIs to convey the precision of adjusted prevalence estimates.^28,29^ Analyses were conducted using Stata statistical software, 15.1 (StataCorp LLC).^30^

### Post Hoc Analysis

To assess how often patients had both conditions with potential benefits from cannabis use and conditions with potential risks, we defined 4 mutually exclusive groups of patients based on their health conditions: (1) only conditions with potential benefits, (2) only conditions with potential risks, (3) both conditions with potential benefits and conditions with potential risks, and (4) neither. Adjusted multinomial logistic regression models were used to estimate the prevalence and 95% CI of these 4 groups across EHR-documented medical, other, and no use.

## Results

A total of 185 565 patients (mean [SD] age, 52.0 [18.1] years; 59% female, 73% White, 94% non-Hispanic, and 61% commercially insured) were screened for cannabis use in a primary care visit during the study period. A total of 3551 patients (2%) had EHR-documented medical cannabis use, 36 599 (20%) had other documented cannabis use, and 145 415 (78%) had no documented cannabis use. Among 40 150 patients with any EHR-documented cannabis use, 9% had medical use documented. Table 1 describes the patient characteristics across categories of EHR-documented cannabis use; unadjusted prevalence estimates of health conditions and selected prescribed medications are given in eTables 1 and 2 in the Supplement.

**Table 1.** Demographic Characteristics of the 185 565 Primary Care Patients in the Study Sample^a^

Characteristic	EHR-documented medical cannabis use (n = 3551)^b^	EHR-documented other cannabis use (n = 36 599)^b^	No EHR-documented cannabis use (n = 145 415)^b^
Sex^c^			
Female	2086 (58.7)	18 771 (51.3)	88 141 (60.6)
Male	1465 (41.3)	17 828 (48.7)	57 272 (39.4)
Age at cannabis screen, y			
18-29	667 (18.8)	10 744 (29.4)	15 350 (10.6)
30-44	777 (21.9)	10 759 (29.4)	27 515 (18.9)
45-64	1187 (33.4)	10 994 (30.0)	56 964 (39.2)
≥65	920 (25.9)	4102 (11.2)	45 586 (31.3)
Race			
American Indian or Alaska Native	50 (1.4)	308 (0.8)	1032 (0.7)
Asian	85 (2.4)	1424 (3.9)	16 137 (11.1)
Black	156 (4.4)	1756 (4.8)	6737 (4.6)
Native Hawaiian or Pacific Islander	19 (0.5)	281 (0.8)	1375 (0.9)
White	2850 (80.3)	28 145 (76.9)	105 156 (72.3)
Multiracial	158 (4.4)	1474 (4.0)	3577 (2.5)
Other or unknown	233 (6.6)	3211 (8.8)	11 401 (7.8)
Hispanic ethnicity	209 (5.9)	2210 (6.0)	8274 (5.7)
Insurance			
Commercial	1838 (51.8)	26 321 (71.9)	84 889 (58.4)
Medicare	1145 (32.2)	4570 (12.5)	46 297 (31.8)
Subsidized or Medicaid	463 (13.0)	4336 (11.8)	11 058 (7.6)
Unknown	105 (3.0)	1372 (3.7)	3171 (2.2)

Abbreviation: EHR, electronic health record.

^a^ Data are presented as number (percentage) of patients unless otherwise indicated.

^b^ Mutually exclusive categories based on practitioner-documented medical cannabis use in the past year (from natural language processing–assisted EHR review and *International Statistical Classification of Diseases and Related Health Problems, Tenth Revision* diagnoses) and patient report of cannabis use in the past year on a screen (see text).

^c^ Sex was missing for 2 patients who had no EHR-documented cannabis use.

In adjusted analyses, patients with EHR-documented medical cannabis use tended to have a higher prevalence of any health conditions for which cannabis use had potential benefits (49.8%; 95% CI, 48.3%-51.3%) compared with patients with other use (39.9%; 95% CI, 39.4%-40.3%) and patients with no use (40.0%; 95% CI, 39.8%-40.2%) (Table 2). Notably, 35.4% (95% CI, 34.1%-36.7%) of patients with medical cannabis use had chronic pain compared with 28.3% (95% CI, 27.8%-28.7%) of patients with other use and 28.3% (95% CI, 28.1%-28.5%) of patients with no use.

**Table 2.** Adjusted Prevalence of Health Conditions, Categorized Based on NASEM Review Among 185 565 Primary Care Patients in the Past Year^a^

Health condition	Prevalence, % (95% CI)		
	EHR-documented medical cannabis use (n = 3551)^b^	EHR-documented other cannabis use (n = 36 599)^b^	No EHR-documented cannabis use (n = 145 415)^b^
Conditions for which cannabis use has potential benefits based on NASEM review^c^			
Any condition	49.8 (48.3-51.3)	39.9 (39.4-40.3)	40.0 (39.8-40.2)
Pain, chronic noncancer^d^^,^^e^	35.4 (34.1-36.7)	28.3 (27.8-28.7)	28.3 (28.1-28.5)
Multiple sclerosis^e^	0.6 (0.4-0.8)	0.4 (0.3-0.5)	0.3 (0.3-0.4)
Muscle spasms or spasticity^e^	5.1 (4.5-5.7)	3.5 (3.3-3.7)	3.5 (3.4-3.6)
Severe nausea^e^	7.6 (6.9-8.2)	4.8 (4.6-5.1)	4.3 (4.2-4.4)
Sleep disorder	21.8 (20.6-22.9)	18.1 (17.7-18.5)	18.5 (18.3-18.6)
Conditions for which cannabis use has potential risks based on NASEM review^c^			
Any condition	60.7 (59.0-62.3)	50.5 (50.0-51.0)	42.7 (42.4-42.9)
Mental health disorders, select	36.2 (34.7-37.6)	26.5 (26.1-27.0)	18.6 (18.4-18.8)
Depressive disorder	33.5 (32.1-34.9)	25.2 (24.7-25.6)	17.7 (17.5-17.9)
Serious mental illness^f^	2.8 (2.4-3.2)	2.0 (1.9-2.1)	1.3 (1.2-1.3)
Respiratory conditions	26.0 (24.7-27.3)	27.5 (27.1-28.0)	28.1 (27.9-28.3)
Bronchitis	15.1 (14.1-16.2)	17.2 (16.8-17.5)	18.6 (18.4-18.8)
COPD	15.6 (14.6-16.6)	15.3 (14.9-15.7)	14.7 (14.5-14.8)
Substance use disorder	21.9 (20.6-23.1)	14.1 (13.7-14.5)	7.1 (7.0-7.3)
Cannabis use disorder	5.9 (5.2-6.6)	1.2 (1.1-1.3)	0.1 (0.1-0.1)
Tobacco use disorder	11.5 (10.5-12.4)	9.7 (9.4-10.0)	5.0 (4.9-5.1)
Alcohol use disorder	4.7 (4.1-5.3)	3.9 (3.7-4.2)	1.9 (1.9-2.0)
Stimulant use disorder	0.6 (0.4-0.7)	0.4 (0.3-0.4)	0.1 (0.1-0.2)
Opioid use disorder	1.9 (1.6-2.2)	1.1 (1.0-1.2)	0.7 (0.7-0.8)
Other drug use disorder	0.9 (0.7-1.1)	0.5 (0.5-0.6)	0.2 (0.2-0.3)
Opioid overdose	0.2 (0.1-0.3)	0.2 (0.1-0.2)	0.1 (0.1-0.1)
Conditions for which cannabis use has inconclusive evidence of benefit or risk based on NASEM review^c^			
Anorexia or cachexia^e^	2.8 (2.4-3.3)	2.0 (1.8-2.2)	1.5 (1.5-1.6)
Cancer^e^	7.4 (6.7-8.1)	7.1 (6.8-7.5)	7.5 (7.4-7.6)
Diabetes type 2	9.0 (8.2-9.7)	9.9 (9.5-10.2)	11.8 (11.6-11.9)
Eating disorder^e^	0.4 (0.3-0.6)	0.3 (0.3-0.4)	0.2 (0.2-0.2)
Epilepsy or seizures^e^	1.3 (1.0-1.6)	1.1 (1.0-1.2)	1.0 (1.0-1.1)
Glaucoma^e^	3.4 (2.9-3.9)	3.9 (3.7-4.2)	4.9 (4.8-5.0)
Heart disease	11.0 (10.2-11.8)	11.9 (11.5-12.3)	13.4 (13.2-13.5)
Hepatitis C^e^	1.0 (0.7-1.3)	0.7 (0.6-0.8)	0.3 (0.3-0.4)
HIV or AIDS^e^	0.3 (0.2-0.4)	0.6 (0.5-0.6)	0.2 (0.2-0.3)
Hypertension	27.7 (26.4-29.0)	28.2 (27.7-28.6)	29.7 (29.5-29.9)
Mental health disorders, select	30.8 (29.4-32.1)	20.2 (19.8-20.6)	14.4 (14.2-14.6)
ADHD	2.3 (1.9-2.7)	2.1 (1.9-2.2)	1.4 (1.3-1.4)
Anxiety	28.7 (27.3-30.0)	18.5 (18.1-18.9)	13.2 (13.0-13.4)
Posttraumatic stress disorder^e^	3.2 (2.7-3.6)	1.5 (1.4-1.6)	0.9 (0.8-0.9)
Renal failure, chronic^e^	0.1 (0.0-0.1)	0.2 (0.1-0.3)	0.2 (0.2-0.2)
Traumatic brain injury^e^	0.8 (0.5-1.0)	0.6 (0.6-0.7)	0.6 (0.6-0.7)

Abbreviations: ADHD, attention-deficit/hyperactivity disorder; COPD, chronic obstructive pulmonary disease; EHR, electronic health record; NASEM, National Academies of Sciences, Engineering, and Medicine.

^a^ Estimates adjusted for age, sex, race, ethnicity, insurance, and note-days.

^b^ Mutually exclusive categories based on practitioner-documented medical cannabis use in the past year (from natural language processing–assisted EHR review and *International Statistical Classification of Diseases and Related Health Problems, Tenth Revision* [*ICD-10*] diagnoses) and patient report of cannabis use in the past year on a screen (see text).

^c^ Categories of health conditions are based on the NASEM 2017 review of the health effects of cannabis and cannabinoids.^6^ Conditions for which cannabis use has potential benefits or risks may be for certain subgroups of patients (eg, cannabis may benefit patients with chemotherapy-induced severe nausea).

^d^ Chronic noncancer pain defined by 2 or more *ICD-10* codes for similar pain types 30 days or more apart or by *ICD-10* codes for general pain.

^e^ Condition authorized for medical cannabis use in Washington State.

^f^ Serious mental illness includes bipolar disorder, schizophrenia, and other psychosis.

Patients with EHR-documented medical cannabis use also tended to have a higher adjusted prevalence of health conditions with potential risks (60.7%; 95% CI, 59.0%-62.3%) compared with patients with other use (50.5%; 95% CI, 50.0%-51.0%) and patients with no use (42.7%; 95% CI, 42.4%-42.9%) (Table 2). Notably, 33.5% (95% CI, 32.1%-34.9%) of patients with medical cannabis use had depression compared with 25.2% (95% CI, 24.7%-25.6%) of patients with other use and 17.7% (95% CI, 17.5%-17.9%) of patients with no use. Similarly, the prevalence of tobacco use disorder was higher for those with medical cannabis use (11.5%; 95% CI, 10.5%-12.4%) compared with those with other use (9.7%; 95% CI, 9.4%-10.0%) and no use (5.0%; 95% CI, 4.9%-5.1%). An exception to this pattern was seen for bronchitis, with 15.1% (95% CI, 14.1%-16.2%) of patients with medical cannabis use having bronchitis compared with 17.2% (95% CI, 16.8%-17.5%) of patients with other use and 18.6% (95% CI, 18.4%-18.8%) of patients with no use.

The adjusted prevalence of conditions with inconclusive evidence of benefit or risk from cannabis varied across EHR-documented cannabis use categories (Table 2). Patients with medical cannabis use had a substantially higher prevalence of anxiety (28.7%; 95% CI, 27.3%-30.0%) compared with patients with other use (18.5%; 95% CI, 18.1%-18.9%) and no use (13.2%; 95% CI, 13.0%-13.4%). Sensitivity analyses adjusting for clinic found similar associations for health conditions across categories of EHR-documented cannabis use (eTable 3 in the Supplement).

**Figure 2.**
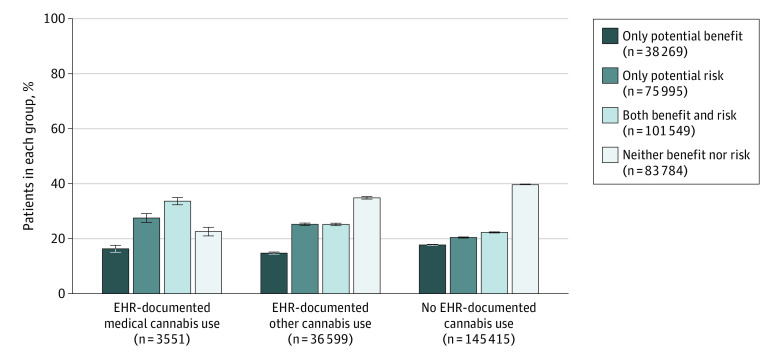
Percentage of the 185 565 Primary Care Patients Screened for Cannabis From November 1, 2017, to October 31, 2018, Who Had Only Health Conditions With Benefit, Only Health Conditions With Risk, Both, or Neither Across Categories of EHR-Documented Past-Year Cannabis Use Estimates were adjusted for age, sex, race, ethnicity, insurance, and note-days. Error bars represent 95% CIs. EHR indicates electronic health record.

Post hoc analyses to describe the proportion of patients who have both health conditions for which cannabis has potential benefits and conditions for which it poses potential risks (Figure 2) indicate that patients with medical cannabis use had a higher prevalence of having both types of conditions (33.6%; 95% CI, 32.3%-34.9%) compared with patients with other use (25.2%; 95% CI, 24.7%-25.6%) and patients with no use (22.3%; 95% CI, 22.1%-22.5%).

Patients with EHR-documented medical cannabis use also had a higher prevalence of selected prescribed medications (54.6%; 95% CI, 53.0%-56.29%) compared with patients with other use (44.4%; 95% CI, 43.9%-44.8%) and patients with no use (37.3%; 95% CI, 37.1%-37.5%) (Table 3). Sensitivity analyses adjusting for clinic found similar associations for prescribed medications across cannabis use categories (eTable 4 in the Supplement).

**Table 3.** Adjusted Prevalence of Selected Prescription Medication Use Among 185 565 Primary Care Patients in the Past Year^a^

Medication use	Prevalence, % (95% CI)		
	EHR-documented medical cannabis use (n = 3551)^b^	EHR-documented other cannabis use (n = 36 599)^b^	No EHR-documented cannabis use (n = 145 415)^b^
Any of the selected medications^c^	54.6 (53.0-56.2)	44.4 (43.9-44.8)	37.3 (37.1-37.5)
Medications that treat conditions for which cannabis may have benefit			
Any	23.6 (22.4-24.7)	16.6 (16.2-17.0)	14.1 (14.0-14.3)
Antiemetics	9.0 (8.3-9.7)	6.5 (6.3-6.8)	6.0 (5.9-6.1)
Muscle relaxants	9.9 (9.2-10.7)	7.9 (7.6-8.2)	6.4 (6.3-6.5)
Medication for neuropathic pain	8.9 (8.3-9.6)	5.8 (5.5-6.1)	4.5 (4.4-4.6)
Medications that treat mental health or substance use disorders			
Any	28.6 (27.3-29.9)	22.2 (21.7-22.6)	16.5 (16.4-16.7)
Antidepressants	27.1 (25.9-28.4)	21.5 (21.0-21.9)	16.0 (15.8-16.1)
Buprenorphine	0.6 (0.5-0.8)	0.6 (0.5-0.7)	0.6 (0.6-0.7)
Naloxone	0.5 (0.4-0.7)	0.2 (0.2-0.3)	0.2 (0.1-0.2)
Naltrexone	0.6 (0.5-0.8)	0.3 (0.3-0.4)	0.2 (0.2-0.2)
Other AUD medications^d^	0.5 (0.3-0.7)	0.4 (0.4-0.5)	0.2 (0.2-0.2)
Medications that are potentially addictive			
Any	34.9 (33.6-36.3)	29.1 (28.7-29.6)	24.3 (24.1-24.5)
Benzodiazepines	11.3 (10.5-12.2)	9.0 (8.6-9.3)	6.5 (6.3-6.6)
Opioids or codeine	26.3 (25.2-27.5)	21.9 (21.5-22.3)	18.9 (18.7-19.1)
Other sedative hypnotics	0.3 (0.2-0.5)	0.4 (0.3-0.4)	0.3 (0.2-0.3)
Stimulants	2.1 (1.8-2.5)	2.5 (2.3-2.6)	1.8 (1.7-1.8)
Z-drugs (for sleep)^e^	1.6 (1.2-1.9)	1.8 (1.7-2.0)	1.3 (1.3-1.4)

Abbreviations: AUD, alcohol use disorder; EHR, electronic health record.

^a^ Estimates adjusted for age, sex, race, ethnicity, insurance, and note-days.

^b^ Mutually exclusive categories based on practitioner-documented medical cannabis use in the past year (from natural language processing–assisted EHR review and *International Statistical Classification of Diseases and Related Health Problems, Tenth Revision* diagnoses) and patient report of cannabis use in the past year on a screen (see text).

^c^ Any prescription medication is an indicator of any filled prescription medications reported in the table.

^d^ Other AUD medications includes acamprosate and disulfiram.

^e^ Z-drugs include zaleplon, zolpidem, and eszopiclone.

## Discussion

This cross-sectional study estimated the prevalence of past-year medical cannabis use, as documented by medical practitioners, among primary care patients routinely screened for past-year cannabis use. In this population, 2% had practitioner documentation of medical cannabis use, and 20% had patient-reported cannabis use without documentation of medical use. Among patients with any past-year cannabis use, 9% had documentation of medical use. Patients with medical cannabis use were more likely than other patients to have health conditions with potential benefits from cannabis use and conditions with potential risks from cannabis use (34% had both). Among patients with medical cannabis use documented, the prevalence of health conditions with potential risks from cannabis use (61%) exceeded that of conditions with potential benefits (50%).

This is the first study, to our knowledge, to estimate the prevalence of EHR-documented medical cannabis use among primary care patients. Prior estimates of the prevalence of medical cannabis use vary, depending on the setting and population of interest, as well as the definition and measure of medical cannabis use. These findings are consistent with those in national surveys that estimate the prevalence of past-year medical cannabis use to be 1% to 2% of US adults^2,31^ or 9% to 12% of US adults who use cannabis.^2,32^ A higher prevalence of past-year medical cannabis use has been observed in primary care samples recruited for trials (15%),^33^ among patients from specialty settings (8%-15%),^34,35,36,37^ and among patients with specific health conditions (2%-30%).^37,38,39,40,41^ This study ascertains medical cannabis use in primary care patients based on their EHR. Although this likely underestimates the true prevalence of medical cannabis use, it accurately reflects practitioner documentation of medical cannabis use, a potentially important part of high-quality primary care.

Prior research^42,43,44,45,46^ has found that patients report using cannabis to address a variety of health conditions. For many of these conditions, there is limited evidence of effectiveness and evidence of risks associated with use.^6,47^ Although studies^6,21,47^ suggest that cannabis use may help patients manage neuropathic pain, improve multiple sclerosis spasticity symptoms, and improve short-term sleep outcomes for some individuals with chronic conditions, more research is needed to understand whether cannabis use can improve these and other physical and mental health symptoms for which current evidence is limited, inconclusive, mixed, or unavailable.^6^ Furthermore, frequent cannabis use has been associated with mental health symptoms and development of substance use disorders, and smoked cannabis may be associated with poorer pulmonary function.^6,23^ The high prevalence of mental health disorders in patients using medical cannabis in this study is concerning because cannabis withdrawal can manifest as increasing anxiety, nervousness, irritability, and depressed mood, and patients with mental health conditions have greater risk of cannabis use disorder.^48^

In light of limited or conflicting evidence on the safety and effectiveness of medical cannabis, health care practitioners may not know how to advise patients who disclose cannabis use for medical or other reasons.^49,50^ Practitioner training about potential risks and benefits of medical cannabis use could prepare practitioners for complex decision-making and support improved primary care for patients with medical cannabis use.^50^ Identifying and documenting medical cannabis use and offering patients information from epidemiologic research on how to minimize risks of cannabis use^51,52,53^ could ensure that patients are aware of risks as well as potential benefits of medical cannabis use.

### Limitations

This study has important limitations. First, the measure of EHR-documented past-year cannabis use may not capture all patients who use cannabis for medical or other reasons because practitioners may not ask about cannabis use, and some patients may not disclose cannabis use. In addition, practitioners may decide not to document medical cannabis use in the EHR (eg, if it is not deemed medically relevant), or they may document cannabis use without patient-reported medical reasons for use. Second, the definition of EHR-documented medical cannabis use used in this study differed from that in others,^2,31,32,33,34,35,36,37,38,39,40,41^ and some mentions of cannabis use may have been misclassified as medical or other during NLP-assisted manual review. Third, this cross-sectional study cannot determine whether patients are using cannabis or not using cannabis because of specific health conditions, whether their cannabis use is having any benefits or harms, or whether differences in adjusted prevalence estimates between groups could reflect confounding by unmeasured factors. Qualitative and mixed methods research may be needed to understand patient-reported reasons for using medical cannabis, and rigorous trials are needed to examine the therapeutic efficacy of cannabis use for each indication and short- and long-term adverse effects in diverse samples. Fourth, primary care patients in this study were predominantly older and White and from a single integrated health system in Washington State. Findings may not generalize to other primary care populations and settings, particularly in states where medical and recreational cannabis are not legal and widely available for adult purchase or where health systems do not routinely ask all primary care patients about cannabis use.

## Conclusions

In a health system that routinely screens primary care patients for cannabis use, approximately 2% of patients (9% of those using cannabis) had EHR-documented medical cannabis use. Patients with medical cannabis use had a high prevalence of health conditions associated with potential risks as well as those associated with potential benefits of cannabis use. These findings suggest that medical practitioners need to be prepared to identify medical cannabis use and discuss potential risks and benefits with their patients.

## Notes

Supplement. **eTable 1.** Unadjusted Prevalence of Health Conditions, Categorized Based on NASEM Review Among 185,565 Primary Care Patients in the Past Year **eTable 2.** Unadjusted Prevalence of Selected Prescription Medications Among 185,565 Primary Care Patients in the Past Year **eTable 3.** Sensitivity Analyses of Additionally Adjusted Prevalence of Health Conditions, Categorized Based on NASEM Review Among 185,565 Primary Care Patients in the Past Year **eTable 4.** Sensitivity Analysis of Additionally Adjusted Prevalence of Selected Prescription Medications Among 185,565 Primary Care Patients in the Past Year
