# Supplementary material for: Prevalence of Medical Cannabis Use and Associated Health Conditions Documented in Electronic Health Records Among Primary Care Patients in Washington State
Source: JAMA Netw Open. 2021 May 6;4(5):e219375. doi: 10.1001/jamanetworkopen.2021.9375 (PMC8103224; doi:10.1001/jamanetworkopen.2021.9375)
Supplement: Supplement. — eTable 1. Unadjusted Prevalence of Health Conditions, Categorized Based on NASEM Review Among 185,565 Primary Care Patients in the Past Year eTable 2. Unadjusted Prevalence of Selected Prescription Medications Among 185,565 Primary Care Patients in the Past Year eTable 3. Sensitivity Analyses of Additionally Adjusted Prevalence of Health Conditions, Categorized Based on NASEM Review Among 185,565 Primary Care Patients in the Past Year eTable 4. Sensitivity Analysis of Additionally Adjusted Prevalence of Selected Prescription Medications Among 185,565 Primary Care Patients in the Past Year [file jamanetwopen-e219375-s001.pdf]

Supplementary Online Content

Matson TE, Carrell DS, Bobb JF, et al. Prevalence of medical cannabis use and associated health conditions documented in electronic health records among primary care patients in Washington State. *JAMA Netw Open*. 2021;4(5):e219375. doi:10.1001/jamanetworkopen.2021.9375

eTable 1. Unadjusted Prevalence of Health Conditions, Categorized Based on NASEM Review Among 185,565 Primary Care Patients in the Past Year

eTable 2. Unadjusted Prevalence of Selected Prescription Medications Among 185,565 Primary Care Patients in the Past Year

eTable 3. Sensitivity Analyses of Additionally Adjusted Prevalence of Health Conditions, Categorized Based on NASEM Review Among 185,565 Primary Care Patients in the Past Year

eTable 4. Sensitivity Analysis of Additionally Adjusted Prevalence of Selected Prescription Medications Among 185,565 Primary Care Patients in the Past Year

This supplementary material has been provided by the authors to give readers additional information about their work.

© 2021 Matson TE et al. *JAMA Network Open.*

eTable 1. Unadjusted Prevalence of Health Conditions, Categorized Based on NASEM Review Among 185,565 Primary Care Patients in the Past Year

Health condition		EHR-documented medical cannabis use[a] (n = 3,551)	EHR-documented other cannabis use[a] (n = 36,599)	No EHR-documented cannabis use[a] (n = 145,415)
		N (%)	*N (%)*	*N (%)*
**Conditions for which cannabis use has potential benefit based on NASEM review**				
Any condition		2,299 (64.7)	11,670 (31.9)	60,509 (41.6)
Pain, chronic non-cancer[b]*		1,854 (52.2)	7,690 (21.0)	43,207 (29.7)
Multiple sclerosis*		44 (1.2)	113 (0.3)	515 (0.4)
Muscle spasms or spasticity*		268 (7.5)	1,062 (2.9)	5,261 (3.6)
Severe nausea*		495 (13.9)	1,583 (4.3)	6,341 (4.4)
Sleep disorder		1,089 (30.7)	5,020 (13.7)	28,192 (19.4)
**Conditions for which cannabis use has potential risks based on NASEM review**				
Any condition		2,544 (71.6)	17,627 (48.2)	62,456 (43.0)
Mental health disorders, select		1,240 (34.9)	9,212 (25.2)	41,436 (28.5)
	Depressive disorder	730 (20.6)	6,092 (16.6)	27,079 (18.6)
	Serious mental illness[c]	797 (22.4)	4,584 (12.5)	22,060 (15.2)
Respiratory conditions		1,737 (48.9)	9,196 (25.1)	27,054 (18.6)
	Bronchitis	1,625 (45.8)	8,670 (23.7)	25,843 (17.8)
	COPD	188 (5.3)	770 (2.1)	1,752 (1.2)
Substance use disorder		1,027 (28.9)	5,001 (13.7)	10,372 (7.1)
	Cannabis use disorder	332 (9.3)	565 (1.5)	112 (0.1)
	Tobacco use disorder	524 (14.8)	3,423 (9.4)	7,256 (5.0)
	Alcohol use disorder	250 (7.0)	1,374 (3.8)	2,802 (1.9)
	Stimulant use disorder	46 (1.3)	174 (0.5)	189 (0.1)
	Opioid use disorder	173 (4.9)	369 (1.0)	1,041 (0.7)
	Other drug use disorder	73 (2.1)	218 (0.6)	311 (0.2)
Opioid overdose		24 (0.7)	35 (0.1)	163 (0.1)
**Conditions for which cannabis use has inconclusive evidence of benefit or risk based on NASEM review**				
Anorexia or cachexia[d]*		171 (4.8)	526 (1.4)	2,376 (1.6)
Cancer*		379 (10.7)	1,594 (4.4)	11,865 (8.2)
Diabetes, type II		457 (12.9)	2,198 (6.0)	18,650 (12.8)
Eating disorder*		30 (0.8)	154 (0.4)	275 (0.2)
Epilepsy or seizures*		87 (2.5)	356 (1.0)	1,460 (1.0)
Glaucoma*		153 (4.3)	707 (1.9)	7,962 (5.5)
Heart disease		604 (17.0)	2,550 (7.0)	21,198 (14.6)
Hepatitis C*		57 (1.6)	176 (0.5)	497 (0.3)
HIV or AIDS*		20 (0.6)	202 (0.6)	328 (0.2)
Hypertension		1,156 (32.6)	6,485 (17.7)	46,939 (32.3)
Mental Health Disorders, select		1,512 (42.6)	8,066 (22.0)	20,181 (13.9)
	ADHD	142 (4.0)	1,077 (2.9)	1,744 (1.2)
	Anxiety	1,417 (39.9)	7,225 (19.7)	18,618 (12.8)
	Posttraumatic stress disorder*	230 (6.5)	567 (1.5)	1,228 (0.8)

© 2021 Matson TE et al. *JAMA Network Open.*

eTable 1, continued	EHR-Documented Medical Cannabis Use[a] (n = 3,551)	EHR-Documented Other Cannabis Use[a] (n = 36,599)	No EHR-Documented Cannabis Use[a] (n = 145,415)
	N (%)	*N (%)*	*N (%)*
Renal failure, chronic*	8 (0.2)	35 (0.1)	342 (0.2)
Traumatic brain injury*	48 (1.4)	227 (0.6)	879 (0.6)

Abbreviations: ADHD, attention-deficit/hyperactivity disorder; COPD, chronic obstructive pulmonary disease; EHR, electronic health record; NASEM, National Academies of Sciences, Engineering, and Medicine

* Condition authorized for medical cannabis use in Washington state

[a] Mutually exclusive categories based on practitioner-documented medical cannabis use in the past year (from natural language processing-assisted EHR review and *International Statistical Classification of Diseases and Related Health Problems, Tenth Revision* diagnoses) and patient report of cannabis use in the past year on a screen (see text)

[b] Chronic non-cancer pain defined by ≥2 ICD codes for similar pain types ≥30 days apart or by ICD codes for general pain

[c] Serious mental illness includes bipolar disorder, schizophrenia, and other psychosis

[d] Includes wasting syndrome authorized for medical cannabis use in Washington state

Categories of health conditions are based on the NASEM 2017 review of the health effects of cannabis and cannabinoids.[6] Conditions for which cannabis use has potential benefits or risks may be for certain subgroups of patients (e.g. cannabis may benefit patients with chemotherapy-induced severe nausea).

© 2021 Matson TE et al. *JAMA Network Open.*

**eTable 2. Unadjusted Prevalence of Selected Prescription Medications Among 185,565 Primary Care Patients in the Past Year**			
Medication use	**EHR-documented medical cannabis use[a]** (n = 3,551)	**EHR-documented other cannabis use[a]** (n = 36,599)	**No EHR-documented cannabis use[a]** (n = 145,415)
	N (%)	*N (%)*	*N (%)*
Any of the Selected Medications[b]	2463 (69.4)	14410 (39.4)	55356 (38.1)
**Medications that treat conditions for which cannabis may have benefit**			
Any	1316 (37.1)	5107 (14)	21045 (14.5)
Antiemetics	593 (16.7)	2103 (5.7)	8772 (6.0)
Muscle relaxants	595 (16.8)	2560 (7.0)	9378 (6.4)
Nerve pain medication	592 (16.7)	1449 (4.0)	6851 (4.7)
**Medications that treat mental health or substance use disorders**			
Any	1449 (40.8)	7083 (19.4)	24543 (16.9)
Antidepressants	1380 (38.9)	6817 (18.6)	23732 (16.3)
Buprenorphine	50 (1.4)	205 (0.6)	893 (0.6)
Naloxone	42 (1.2)	115 (0.3)	219 (0.2)
Naltrexone	71 (2.0)	80 (0.2)	321 (0.2)
Other AUD medications[c]	33 (0.9)	157 (0.4)	288 (0.2)
**Medications that are potentially addictive**			
Any	1804 (50.8)	9003 (24.6)	36249 (24.9)
Benzodiazepines	672 (18.9)	2671 (7.3)	9670 (6.6)
Opioids or Codeine	1460 (41.1)	6355 (17.4)	28519 (19.6)
Other sedative hypnotics	24 (0.7)	105 (0.3)	380 (0.3)
Stimulants	136 (3.8)	1193 (3.3)	2299 (1.6)
Z-drugs[d] (for sleep)	86 (2.4)	541 (1.5)	2010 (1.4)

Abbreviations: AUD, alcohol use disorder; EHR, electronic health record

[a] Mutually exclusive categories based on practitioner-documented medical cannabis use in the past year (from natural language processing-assisted EHR review and *International Statistical Classification of Diseases and Related Health Problems, Tenth Revision* diagnoses) and patient report of cannabis use in the past year on a screen (see text)

[b] Any prescription medication is an indicator of any filled prescription medications reported in the table

[c] Other AUD medications includes acamprosate and disulfiram

[d] Z-drugs include zaleplon, zolpidem, and eszopiclone

© 2021 Matson TE et al. *JAMA Network Open.*

eTable 3. Sensitivity Analyses of Additionally Adjusted[a] Prevalence of Health Conditions, Categorized Based on NASEM Review Among 185,565 Primary Care Patients in the Past Year			
Health condition	**EHR-documented medical cannabis use[b]** (n = 3,551)	**EHR-documented other cannabis use[b]** (n = 36,599)	**No EHR-documented cannabis use[b]** (n = 145,415)
	Percent (95% CI)	*Percent (95% CI)*	*Percent (95% CI)*
**Conditions for which cannabis use has potential benefit based on NASEM review**			
Any condition	49.7 (48.2-51.2)	40.5 (40.0-41.0)	39.8 (39.6-40.1)
Pain, chronic non-cancer[c]*	35.2 (33.9-36.5)	28.7 (28.2-29.1)	28.2 (28.0-28.4)
Multiple sclerosis*	0.6 (0.4-0.8)	0.4 (0.4-0.5)	0.3 (0.3-0.4)
Muscle spasms or spasticity*	5.1 (4.5-5.7)	3.7 (3.4-3.9)	3.5 (3.4-3.6)
Severe nausea*	7.6 (6.9-8.2)	5 (4.7-5.2)	4.3 (4.2-4.4)
Sleep disorder	21.7 (20.6-22.9)	18.5 (18.1-18.9)	18.4 (18.2-18.6)
**Conditions for which cannabis use has potential risks based on NASEM review**			
Any condition	60.6 (59.0-62.3)	51.0 (50.5-51.5)	42.6 (42.3-42.8)
Mental health disorders, select	36.2 (34.7-37.6)	26.5 (26.1-27)	18.6 (18.4-18.8)
Depressive disorder	33.5 (32.1-34.9)	25.2 (24.8-25.7)	17.7 (17.5-17.9)
Serious mental illness[d]	2.8 (2.4-3.3)	2.0 (1.8-2.1)	1.3 (1.2-1.3)
Respiratory conditions	26.0 (24.7-27.3)	28.0 (27.5-28.5)	28 (27.8-28.2)
Bronchitis	15.2 (14.1-16.2)	17.4 (17.0-17.8)	18.6 (18.4-18.8)
COPD	15.5 (14.5-16.5)	15.6 (15.2-16.0)	14.6 (14.4-14.8)
Substance use disorder	21.6 (20.4-22.8)	14.5 (14.2-14.9)	7.1 (7.0-7.2)
Cannabis use disorder	5.9 (5.2-6.6)	1.3 (1.2-1.4)	0.1 (0.1-0.1)
Tobacco use disorder	11.3 (10.4-12.2)	10.2 (9.8-10.5)	4.9 (4.8-5.0)
Alcohol use disorder	4.6 (4.0-5.2)	3.9 (3.7-4.1)	1.9 (1.9-2.0)
Stimulant use disorder	0.6 (0.4-0.7)	0.4 (0.3-0.4)	0.1 (0.1-0.2)
Opioid use disorder	1.9 (1.6-2.2)	1.1 (1.0-1.3)	0.7 (0.7-0.8)
Other drug use disorder	0.9 (0.7-1.1)	0.5 (0.5-0.6)	0.2 (0.2-0.3)
Opioid Overdose	0.2 (0.1-0.3)	0.2 (0.1-0.2)	0.1 (0.1-0.1)
**Conditions for which cannabis use has inconclusive evidence of benefit or risk based on NASEM review**			
Anorexia or cachexia[e]*	2.8 (2.4-3.3)	2.0 (1.9-2.2)	1.5 (1.5-1.6)
Cancer*	7.5 (6.7-8.2)	7.1 (6.8-7.4)	7.5 (7.4-7.6)
Diabetes, type II	8.9 (8.1-9.6)	10.2 (9.9-10.6)	11.7 (11.6-11.9)
Eating disorder*	0.4 (0.3-0.5)	0.3 (0.3-0.4)	0.2 (0.2-0.2)
Epilepsy or seizures*	1.3 (1.0-1.6)	1.1 (1.0-1.2)	1.0 (0.9-1.1)
Glaucoma*	3.4 (2.9-4.0)	3.9 (3.6-4.1)	4.9 (4.8-5)
Heart disease	11.1 (10.3-11.8)	12.1 (11.8-12.5)	13.3 (13.2-13.5)
Hepatitis C*	1.0 (0.7-1.3)	0.7 (0.6-0.8)	0.3 (0.3-0.4)
HIV or AIDS*	0.3 (0.2-0.4)	0.4 (0.4-0.5)	0.2 (0.2-0.3)
Hypertension	27.7 (26.4-28.9)	28.8 (28.3-29.3)	29.6 (29.4-29.7)
Mental health disorders, select	30.8 (29.5-32.2)	20.2 (19.8-20.6)	14.4 (14.2-14.6)
ADHD	2.3 (1.9-2.7)	2.0 (1.9-2.1)	1.4 (1.3-1.5)
Anxiety	28.7 (27.4-30)	18.5 (18.2-18.9)	13.2 (13.0-13.4)
Posttraumatic stress disorder*	3.1 (2.7-3.5)	1.5 (1.4-1.6)	0.9 (0.8-0.9)

© 2021 Matson TE et al. *JAMA Network Open.*

eTable 3, continued	EHR-Documented Medical Cannabis Use[b] (n = 3,551)	EHR-Documented Other Cannabis Use[b] (n = 36,599)	No EHR-Documented Cannabis Use[b] (n = 145,415)
	Percent (95% CI)	*Percent (95% CI)*	*Percent (95% CI)*
Renal failure, chronic*	0.1 (0.0-0.1)	0.2 (0.1-0.3)	0.2 (0.2-0.2)
Traumatic brain injury*	0.8 (0.5-1.0)	0.6 (0.6-0.7)	0.6 (0.6-0.7)

Abbreviations: ADHD, attention-deficit/hyperactivity disorder; COPD, chronic obstructive pulmonary disease; EHR, electronic health record; NASEM, National Academies of Sciences, Engineering, and Medicine

* Condition authorized for medical cannabis use in Washington state

[a] Estimates adjusted for age, sex, race, ethnicity, insurance, note-days, and clinic

[b] Mutually exclusive categories based on practitioner-documented medical cannabis use in the past year (from natural language processing-assisted EHR review and *International Statistical Classification of Diseases and Related Health Problems, Tenth Revision* diagnoses), and patient report of cannabis use in the past year on a screen (see text)

[c] Chronic non-cancer pain defined by ≥2 ICD codes for similar pain types ≥30 days apart or by ICD codes for general pain

[d] Serious mental illness includes bipolar disorder, schizophrenia, and other psychosis

[e] Includes wasting syndrome authorized for medical cannabis use in Washington state

Categories of health conditions are based on the NASEM 2017 review of the health effects of cannabis and cannabinoids.[6] Conditions for which cannabis use has potential benefits or risks may be for certain subgroups of patients (e.g. cannabis may benefit patients with chemotherapy-induced severe nausea).

© 2021 Matson TE et al. *JAMA Network Open.*

**eTable 4. Sensitivity Analysis of Additionally Adjusted[a] Prevalence of Selected Prescription Medications Among 185,565 Primary Care Patients in the Past Year**			
	**EHR-documented medical use[b]** (n = 3,551)	**EHR-documented other use[b]** (n = 36,599)	**No EHR-documented cannabis use[b]** (n = 145,415)
	Percent (95% CI)	*Percent (95% CI)*	*Percent (95% CI)*
Any of the selected medications[c]	54.5 (53.0-56.1)	45.0 (44.5-45.5)	37.1 (36.9-37.4)
**Medications that treat conditions for which cannabis may have benefit**			
Any	23.5 (22.4-24.7)	17.1 (16.7-17.5)	14.1 (13.9-14.2)
Antiemetics	9.0 (8.3-9.7)	6.7 (6.4-7.0)	6.0 (5.8-6.1)
Muscle relaxants	9.9 (9.1-10.7)	8.2 (7.9-8.5)	6.3 (6.2-6.4)
Nerve pain medication	9.0 (8.3-9.7)	6.0 (5.7-6.3)	4.4 (4.3-4.5)
**Medications that treat mental health or substance use disorders**			
Any	28.6 (27.3-29.9)	22.3 (21.9-22.8)	16.5 (16.3-16.7)
Antidepressants	27.2 (25.9-28.5)	21.6 (21.2-22.1)	15.9 (15.8-16.1)
Buprenorphine	0.6 (0.5-0.8)	0.6 (0.5-0.7)	0.6 (0.6-0.7)
Naloxone	0.5 (0.4-0.7)	0.2 (0.2-0.3)	0.2 (0.1-0.2)
Naltrexone	0.6 (0.5-0.8)	0.3 (0.3-0.4)	0.2 (0.2-0.2)
Other AUD medications[d]	0.5 (0.3-0.7)	0.4 (0.3-0.5)	0.2 (0.2-0.2)
**Medications that are potentially addictive**			
Any	34.8 (33.5-36.2)	29.7 (29.2-30.1)	24.2 (24.0-24.4)
Benzodiazepines	11.3 (10.5-12.2)	9.0 (8.7-9.4)	6.4 (6.3-6.6)
Opioids or codeine	26.2 (25.0-27.4)	22.6 (22.1-23)	18.8 (18.6-19)
Other sedative hypnotics	0.3 (0.2-0.5)	0.4 (0.3-0.5)	0.3 (0.2-0.3)
Stimulants	2.2 (1.8-2.5)	2.4 (2.3-2.5)	1.8 (1.7-1.8)
Z-drugs[e] (for sleep)	1.6 (1.2-1.9)	1.8 (1.6-1.9)	1.3 (1.3-1.4)

Abbreviations: AUD, alcohol use disorder; EHR, electronic health record

[a] Estimates adjusted for age, sex, race, ethnicity, insurance, note-days, and clinic

[b] Mutually exclusive categories based on practitioner-documented medical cannabis use in the past year (from natural language processing-assisted EHR review and *International Statistical Classification of Diseases and Related Health Problems, Tenth Revision* diagnoses) and patient report of cannabis use in the past year on a screen (see text)

[c] Any prescription medication is an indicator of any filled prescription medications reported in the table

[d] Other AUD medications includes acamprosate and disulfiram

[e] Z-drugs include zaleplon, zolpidem, and eszopiclone

© 2021 Matson TE et al. *JAMA Network Open.*
